# Qualification of Food Intake by the Roma Population in the Region of South Bohemia

**DOI:** 10.3390/ijerph15020386

**Published:** 2018-02-23

**Authors:** Lenka Sedova, Valerie Tothova, Dita Novakova, Vera Olisarova, Sylva Bartlova, Frantisek Dolak, Alena Kajanova, Radka Prokesova, Vera Adamkova

**Affiliations:** Department of Nursing, Midwifery and Urgent Care, Faculty of Health and Social Studies, University of South Bohemia in Ceske Budejovice, U Vystaviste 26, 37001 Ceske Budejovice, Czech Republic; tothova@zsf.jcu.cz (V.T.); novakovad@zsf.jcu.cz (D.N.); volisarova@zsf.jcu.cz (V.O.); sbartlova@zsf.jcu.cz (S.B.); fdolak@zsf.jcu.cz (F.D.); kajanova@zsf.jcu.cz (A.K.); rprokes@zsf.jcu.cz (R.P.); vead@medicon.cz (V.A.)

**Keywords:** nutritional records, Nutridan, energy intake, Roma, frequency, food

## Abstract

The article presents the results of a correlation study, aimed at quantifying the food intake of the Roma population in the South Bohemian Region. To achieve the goal, we applied the method of one-day dietary recall and frequency food analysis (non-standardized). The quantification was carried out by analysis in the Nutridan program. The study involved 302 Roma persons and 298 persons in the control group. Both groups had the same representation of males and females (50:50). The age categories of both sets differed; the average age of the Roma was lower (39.2 years) (*p* < 0.001). The probands from the Roma population were chosen with the help of the snowball method through known respondents. The statistical analysis shows differences in nutritional estimate between the Roma population and the control sample. The Roma differ in their energy intake. Both groups showed lower intake of sugars, below 50% total energy intake (TEI) and higher intake of fats, above 30% TEI. The respondents from both groups consume little fruits and vegetables, which may be connected with their low dietary fiber intake. In addition to the differences in the nutritional estimates, we recorded statistically significant differences in body mass index (BMI; *p* < 0.001), in age (*p* < 0.001), regular alimentation (*p* = 0) and demanding physical activities (*p* = 0). In spite of the fact our groups differed in age (the Roma are younger), it can be assumed that the obesity of the Roma may be caused by unbalanced alimentation and lack of physical activities.

## 1. Introduction

The connection of health and nutrition was known long time before Christ, as evidenced by the valid rules of Hippocrates’ therapeutic dietics. The current alimentation and dietary suggestions can be found in a number of documents and recommendations [1,2,3].

In 2011, the Společnost pro výživu (Association for Nutrition, SPV) issued a set of dietary recommendations for the Czech Republic together with the recommendations of the German Association for Nutrition (Deutsche Gesellschaft für Ernährung), with the Austrian Association for Nutrition (Österreichische Gesellschaft für Ernährung) and with the Swiss Association for Nutrition (Schweizerische Gesellschaft für Ernährung). Some publications call these the D-A-CH recommendations (an acronym of the countries with common recommendations) [4].

These recommendations were updated by the SPV in 2013. The 2013 recommendations do not differ from the nutritional goals for the European Union (EU) [2]. The Czech recommendations stipulate that the energy intake and expenditure should be regulated in order to maintain a consistent body weight (body mass index (BMI) = 18–25 kg/m^2^). The total food fat intake should amount to 30–35% of total energy intake (TEI). Unsaturated fatty acids (FA) should constitute 10% of TEI (20 g), and polyunsaturated FA are represented by 7–10% of TEI. As for the fat component, 1% (2.5 g) of *trans*-unsaturated FA per day should not be exceeded. The total cholesterol in the diet should not exceed a value of 300 mg/day. Further recommendations are aimed at reduction of simple sugars and reduction of the amount of common salt (5–6 g/day). The recommendations suggest a fiber intake of 30 g/day. Another part discusses the intake of minerals providing for antioxidant and protective processes in the organism. Fruits and vegetables in natural form are the source of minerals [4,5].

The basic food components are divided into two groups, micronutrients and macronutrients. The macronutrients include fat, sugars and proteins. These nutrients are energy carriers. Their recommended proportion in the diet is proteins 15% of TEI, fats 30% of TEI, sugars 55% [6,7].

At present, nobody questions the importance of the diet for the health. A World Health Organization analysis [2] states that the dietary habits damage the population health and wellbeing in all countries of the EU. The diseases related to the nutritional conditions (both malnutrition and overweight or obesity) have high social and economic consequences for individuals, families, communities and governments of the EU countries [8].

Overweight and obesity constitute one of the health problems closely related to the quality and quantity of food intake. As Hainer states, the correlation between the excessive intake of fats and simple sugars and obesity was confirmed by a number of studies, but comprehensive meta-analyses point out also the importance of the amount of the total energy intake that is crucial for the accumulation of fat in the organism [9]. The quality and quantity of food intake are not the only factors leading to overweight and obesity [10]. The social status may be a further factor contributing to overweight and obesity [11]. Socio-economic inequalities and incidence of obesity were discussed for example by the 2007 EUROTHINE study which states that, by estimate, socio-economic status (SES) inequalities may be related with 13% of male obesity and 45% of female obesity [12]. Nutrition is one of the social determinants of health [13].

Selection of foodstuffs with low SES involves three basic factors: price, availability and knowledge. In a narrower context, people with lower SES buy energetically unsuitable foodstuffs more frequently; another factor may consist in insufficient access to kitchen equipment, which leads to the purchase of ready-made meals. Nutritional literacy is another influencing factor. The selection of foodstuffs is often also based on cultural and social influences [14,15].

The Roma constitute the largest ethnically different group in the Czech Republic. The demographic data of the minority are inaccurate [16,17,18,19,20], and relevant data concerning the lifestyle of the Roma in the context of their health are missing in the Czech Republic at present [19]. Nevertheless, some estimates suggest that the Roma minority has a 10-year shorter life expectancy, as compared to the majority population. One of the causes explaining that difference is a distinctive difference in their lifestyle in the context of health, e.g., incidence of smoking, eating habits, physical inactivity, stress [15,19]. In this context the partial results of our correlation study suggest a higher incidence of anthropometric indicators of overweight and obesity among the Roma in the South Bohemian Region [19,20].

## 2. Goals

The goal of the study consisted in describing the prevalence of overweight and obesity among the Roma in the South Bohemian Region (Czech Republic). The goal was achieved under use of multiple research techniques; the purpose consisted in describing internal (genetic) and external (socio-demographic, alimentation and eating habits, physical activities, approach to health) factors leading to obesity among the Roma. One of the goals consisted in ascertaining the differences in eating habits of the Roma and the majority population.

## 3. Materials and Methods

We made use of multiple research methods to achieve our goals. To determine the nutrition status of the Roma, we made use of anthropometric measurements —determination of height, weight, BMI, waistline, and determination of total fat by a bimanual bioimpedance method. To determine the internal factors, we detected differences at DNA level. As for the external factors, we focused on monitoring the eating habits. We used a combination of two techniques for it: the 24 h dietary recall and the frequency foodstuff analysis. To analyze the diet, we had to know the respondent’s gender, height and weight, age and daily physical activities. The height and weight were evaluated under standard conditions with the help of SEGA 213 scales and SEGA 813 height meters. The respondents were weighed and measured in underwear during the day. The body mass index was subsequently determined from the height and weight data.

The data were collected from June 2015 to March 2016. The Roma population was sampled using estimates of the Roma population in the South Bohemian Region. A random proportional sample was obtained based on the Roma population’s distribution in the South Bohemian Region (CZ), as well as on gender.

We collected the data by interviews with each study participant. The study also included a pilot study in which we checked the efficiency of the created record documents and of the individual interview items. The interview, its recording, the anthropometric measurements and DNA smears were collected by general nurses (research team). The data collection was made by the snowball technique in both groups (the Roma set and the control set). The structure of the Roma group by gender was derived from the majority population proportions, where the representation of males and females is assumed to be approximately 50:50. The selected group was structured as representative for the Roma population, with approximately identical representation of the number of males and females. The Roma probands were selected through respondents from all over the South Bohemian Region. The identification of the Roma was based on self-determination of Roma ethnic membership.

The nutritional estimate analysis was performed with the help of the analysis of nutritional records in form of 24 h dietary recall and the questionnaire of frequency foodstuff analysis (non-standardized). As Svačina states, such records can be used in practice [8]. The record contains the diet for the preceding day and the size of the portions is estimated. A trained interviewer filling out the recalled diet and the frequency foodstuff analysis record together with each respondent ensured the validation of the technique. The nutritional records were analyzed by Nutridan II program. Nutridan is a nutritional software (Müllerová Dana, Plzeň, Czech Republic) that evaluates the individual nutrients in relation to gender, age, BMI and physical activities. The data presented in the article are considered already in relation to gender, age and physical activities carried out during the day for which the diet is described. The data are converted into percentage of the total energy intake. To determine the energy estimate, we categorized the daily energy expenditure (PAL) in a range of 1.6–1.7, i.e., for persons with sedentary activities or slightly demanding activities made standing up or walking. The set did not include any respondent carrying out a physically demanding job.

As there are few valid references dealing with the issue of nutrition of the Roma, we compared the quantified results with a general non-Roma population (control group). The nutritional values were quantified in compliance with the current nutritional recommendation for the Czech Republic population and with the updated recommendation of the 2011 Reference Values for Intake of Nutrients of the CZ Association for Nutrition.

Statistic testing was further performed in the SADS (Statistical analysis of social data, Czech Republic) and SPSS 16 programs (IBM Analytics. Armonk, NY, USA). After sorting out incomplete diets (−14), we used 292 diets filled out by the Roma population and 294 diets from the control group.

The statistical connections between the characteristics observed were tested with the help of Pearson χ^2^. The averages were compared by the Student *t*-test and with help of the non-parametric Mann–Whitney U test for data without normal distribution.

## 4. Set

The set of respondents was put together based on the basic goal of the project, i.e., comparison of incidence of overweight and obesity in the Roma and majority populations in the South Bohemian Region. As it was a comparative analysis, the number of the respondents in the individual sets had to be equal. From the perspective of the respondents’ membership of the Roma minority or of the majority population, the selection pool included virtually the same number in both groups, i.e., the Roma minority (302, i.e., 50.3%) and the majority population (298, i.e., 49.7%). For the purpose of evaluation of the diet quality, 14 nutritional records were eliminated because of incompleteness. The eliminated records included 10 nutritional records not completed by Roma individuals and four nutritional records not completed by non-Roma individuals—members of the control group.

The group selection planning was performed with the snowball sampling technique. That technique can be implemented for so called hidden groups, when the structure and distribution of the basic group is unknown, in our case for the Roma population in the South Bohemian Region. The Roma were contracted through a respondent. The contacts were linked up with the help of the snowball technique. When structuring the selected Roma group, we estimated the proportion of males and females to be similar to the majority population. The accurate number of Roma in the Czech Republic is not known.

The structure of the Roma sample was derived from the majority population, as it is assumed that the Roma minority has approximately 50:50 representation of men and women as well. So that indicator can be considered representative. As for gender, the Roma set consisted of 152 men (50.3%) and 150 women (49.7%).

The majority of population was subject to quota selection (also randomly selected with the help of quotas), where the gender was specified as a quota item (50:50). The majority set was representative from the gender perspective. The majority sample included 148 (49.7%) men and 150 (50.3%) women (Table 1).

Age had not been determined as a feature of representativeness, and the structure of the majority population does not match the population structure in the South Bohemian Region from the perspective of age. The distribution of age categories in both sets was unequal(Table 1). The Roma set included prevailing number of respondents of younger age categories—18–29 years 43.8% (132), while the same age category was represented by 28.2% (84) respondents from the majority population. Both sets included relatively few respondents aged 60 and more years.

## 5. Results

In the study participated 302 Roma persons and 298 persons from control group. The sets differed in their age group structure (*p* < 0.001). The largest group in the Roma population was in the 18–29 years range (43.8%, Table 1). From the perspective of number of males and females, the sets were similar. The Roma group included 50.3% (152) males and the control group included 49.7% (148) males. The average age of the Roma group was 39.2 years, while the average age of the control group was 44.6 years.

The purpose of this part of the study was to compare the quantified nutritional values based on the dietary recall data of the Roma and non-Roma populations living in the South Bohemian Region. The ascertained total energy intake results (kcal) have an ambivalent form. The average value of total daily energy income amounts, for the Roma group, was 1900.8 kcal, as against 2519.3 kcal of the control group (Table 2). The difference between the calculated energy intake of the diet of the Roma and of the control set members was evaluated as statistically significant (*p* < 0.001).

The protein content in the daily diet is similar in the Roma minority and in the control group and does not differ in a statistically significant manner. It usually oscillates between 10 and 20% of TEI; the average for the whole set amounted to 17.1% of TEI (Table 2). Statistical differences were not found either when comparing the Roma and non-Roma group (*p* = 0.864; not significant) nor when comparing genders. Optimal fat intake ranging from 25–30% was reported by 16.8% (*n* = 49) of the Roma respondents and by 20.1% (*n* = 59) of the majority respondents. Average fat value of the Roma population amounted to 34.9% TEI and that of the control group amounted to 33.5% TEI (Table 2). The comparison of the averages did not prove any statistical connection (*p* = 0.122).

Average sugar intake of the Roma population amounted to 44.4% TEI and that of the control set to 44.9% TEI. Further testing of differences related to genders and individual groups (*p* = 0.345) does not suggest any statistically significant points. An energy intake amounting to 55–60% of TEI was found in 10.6% (*n* = 31) of the Roma respondents and in 7.8% (*n* = 23) of the majority respondents; most of the respondents from both groups had an intake below 55% TEI.

In the analysis of the diets, we also focused on the amount of consumption of fiber (in grams). The average amount of dietary fiber was 19.5 in the Roma set and 20.2 in the control set (*p* = 0.154). Most respondents had lower consumption of fibers than the recommended amount (30 g).

We calculated the BMI to determine the energy estimate. The average BMI value was 32.3 in the Roma set and 26.0 in the control set. The results show that the Roma population had BMI above 30, i.e., at the obesity level more frequently (*p* < 0.001). The BMI testing in connection to gender suggests a high proportion of obesity in Roma women (*p* < 0.001).

The frequency diet analysis includes the question of intake of fruits and vegetables; a small part of respondents from both groups consume fruits and vegetables several times a day. They consume less vegetables than fruits. Sixty six percent (201) of Roma respondents and 77.5% (231) of respondents from the control set have regular alimentation. The question of execution of demanding physical activities had more positive answers in the control set (211, 70.5%) than in the Roma set (30.7%).

## 6. Discussion

The results found are related to the processing of the grant project (COST—LD 14114) dealing with the description of internal and external factors related to overweight and obesity of the inhabitants of the South Bohemian Region. The results of this article are related to alimentation particularities, specifically to analysis of a 24 h one-day recalled diet. The article was aimed at analyzing the diets in the Roma ethnic group and comparing the results with the control group.

The energy intake is influenced by the representation of individual food components; a number of important studies [9] confirmed a causal relation of the incidence of obesity and of the amount of fats and simple sugars consumed. Šedová, Tóthová and Olišarová published in their reports data from 2016 where a higher incidence of overweight and obesity was found among the Roma [17,18,19,20]. Gecková, a Slovak author, reached similar conclusions [21,22,23]. Our Roma set showed a prevalence of obesity, as compared to the control set—measured by BMI (<0.001). It must be added that the Roma set was younger than the control set (<0.001).

The analysis of the estimated energy intake in the Roma population is ambivalent in our set. As for the Roma respondents, the average daily energy intake amounted to 1900.8 kcal, while the majority population reported 2519.3 kcal. The results are statistically significant (<0.001). The difference may be influenced by considerably higher physical activities in the control set. Sports activities (apart from walk under 30 min) are performed by 71.3% (211) of the approached persons from the control group. A difference in energy estimate was found also in relation to genders.

Increased consumption of fats has a significant share in the increased energy intake [7]. According to the current nutritional recommendations, fats should not constitute 30% of total energy intake [24]. The analysis of the one-day diet showed that the average consumption of fats in the Roma set amounts to 34.9% TEI, while in the control set to 33.5% TEI (*p* = 0.122). As Hainer [9] states, “actually, they constitute 36–38% of the energy intake, and the country population and older persons frequently have more than 40% of fats in their energy intake” [9]. The testing of the averages was not significant (*p* = 0.122).

The consumption of saccharides is significant in the incidence of overweight and obesity. “Increased consumption of simple saccharides, like saccharose and fructose, is related to obesity” [9]. Saccharides may have different effects on the incidence of obesity, according to the glycemic index level. Foodstuffs with low glycemic index lead to lower postprandial increase of blood sugar. The results of saccharide intake show that about 55–60% were consumed by 10.6% (*n* = 31) of the Roma and by 7.8% (*n* = 23) persons from the control group. Our respondents from both groups had lower consumption of saccharides than 50% TEI. The intake of fruits can be related with the intake of sugars. Fruits are consumed several times a day by 4.6% (14) Roma and by 12.1% (36) respondents from the control group. Fruits, like vegetables, contain soluble fibers [6]. The fibers from the fruits and vegetables influence the satiety, as well as the lipid spectrum and the metabolism of saccharides [9]. In our set, vegetables are regularly consumed several times a day by more than 3.6% (11) Roma respondents and by 7.7% (23) respondents from the control group. The low consumption of fruits and vegetables can correlate to the low consumption of fiber. The analysis of fiber consumption shows that a sufficient amount (up to 30 g/day) is consumed by only 10% of the respondents from both sets.

The relation of increased protein intake and obesity remains unprovable so far. An explanation states that people with higher BMI consume more animal proteins, which is accompanied by increased fat intake [9]. The analysis of the 24 h nutritional records shows that there is no statistically significant difference between consumption of proteins in the Roma group and the control group. The most respondents from both groups consume proteins in a range from 10–20% of TEI (Table 2).

Other factors that can influence the incidence of overweight and obesity include the frequency of food intake [6]. In our sets, 73.5% of the respondents eat 3–5 times a day—breakfast, snack, lunch, snack and dinner.

The issue of nutritional estimate is related also to physical activities. At the time of the data collection, the respondents from our field study reported rather light physical activities; their jobs did not involve demanding physical work. Therefore, the question of more demanding physical activities exercised more by respondents from the control group (71.7%) is interesting. In the Roma group, 30% probands reported exercising.

The results show qualitative drawbacks in the nutrition of both exposed groups, highlighting a need to increase the nutritional literacy among the population. Our conclusions may contribute to explain the incidence of overweight and obesity in the Roma population.

## 7. Study Limits

The study limits include the selection of the group of Roma respondents. The selection was made based on claimed membership in the Roma ethnicity. Another limit consists in the recalled diet and its recording, sometimes including difficult agreements about the size of a portion. The selected method of 24 h dietary recall should ideally make records for several days, which was not possible, in view of the educational literacy of the Roma adult population. Another limit consists in the selection of a specific region of the Czech Republic where we focused on only one of the 14 regions.

## 8. Importance for Practice

Our study provides interesting data of internal and external factors of overweight and obesity in the Roma population. The Roma population is the largest ethnically different population in the Czech Republic, with a different socio-demographic description and insufficiently known mortality and morbidity. The health risk factors, which contribute to different mortality and morbidity, are different as well. Our results became a part of the strategic materials of the South Bohemian Region and they were also submitted to the Cabinet Office of the Czech Republic; the regional coordinators for Roma matters were familiarized with the key results too. Thus, the results can contribute to improve the level of community care, to increase the awareness of overweight and obesity among the Roma and to improve the nutritional literacy. A practical outcome of this project consists in an educational material on the principles of healthy alimentation (translated into the Roma language).

## 9. Conclusions

Our analysis of 24 h nutritional records has shown that the Roma respondents exceeded more frequently the reference values for energy intake (as calculated in relation to gender, age and physical activity). Based on our data, it can be assumed that the proportion of fats in the diet of the Roma and of the majority population in the South Bohemian Region is higher than the current nutritional recommendations stipulate. We can assume that, in view of the fact of the lower average age of the Roma, the generally higher average BMI and the energy imbalance (intake and expenditure), we can consider the Roma respondents at risk from the perspective of prevalence of obesity. The results found cannot be generalized however for the eating behavior of the Roma all over the Czech Republic. 

## Figures and Tables

**Table 1:** Age structure of selection set.

	Roma People		Control Set	
	(*n*)	%	(*n*)	%
**18–29 years**	133	43.8	84	28.2
**30–39 years**	49	16.3	78	26.2
**40–49 years**	56	18.6	47	15.8
**50–59 years**	39	13.0	37	12.4
**60–69 years**	20	6.6	31	10.4
**70 and more years**	5	1.7	21	7.0
**Total**	302	100.0	298	100.0

**Table 2:** Basic characteristics of nutritional estimate—as evaluated from diet for last 24 h.

	Roma Population (*N* = 292)			Control Set (*N* = 294)			
	Me	(X)	SD	Me	(X)	SD	*p*-Value
**Age**	39.2	39.2	12.8	39.5	44.5	15.1	<0.001
**BMI**	29.9	32.3	5.6	25	26.0	6.0	<0.001
**Kcal**	1907.6	1900.8	665.6	2525.2	2519.3	764.2	<0.001
**Index Protein % TEI**	16.7	17.1	4.5	17.0	17.1	3.9	0.864
**Index Fat % TEI**	35.3	34.9	9.0	32.9	33.5	8.1	0.122
**Index Saccharides % TEI**	44.8	44.4	9.9	44.31	44.9	9.8	0.345
**Index Fiber (g)**	18.3	19.5	8.6	21.0	20.2	8.7	0.154
**Consumption of fruits—several times a day n_1_/f_i_**	14/4.6	-	-	36/12.1	-	-	0.0
**Consumption of vegetables—several times a day n_1_/f_i_**	11/3.6	-	-	23 /7.7	-	-	0.0
**Regular alimentation—n_1_/f_i_**	201/68.9	-	-	231/78.5	-	-	0.0
**Demanding physical activities than walking n_1_/f_i_**	93/30.8	-	-	211/71.7	-	-	0.0

Me: median; n_i_: absolute frequency; f_i_: relative frequency; (X): average; SD: standard deviation; *p*-value: the test value for categoric data is calculated with the help of χ^2^ test; *t*-test was used for cardinal parametric variable; BMI: body mass index; kcal: energy unit used at describing the energy value of foodstuffs; TEI: total energy intake; g: gram.
